# Rate of detecting CIN3+ among patients with ASC-US using digital colposcopy and dynamic spectral imaging

**DOI:** 10.3892/ol.2020.11878

**Published:** 2020-07-16

**Authors:** Karen Eloise Harris, Philip Todd Lavin, Mark Donnell Akin, Emmanouil Papagiannakis, Sara Denardis

**Affiliations:** 1 Department of Obstetrics and Gynecology, College of Medicine, University of Central Florida, Gainesville, FL 32605, USA; 2 Boston Biostatistics Research Foundation, Framingham, MA 01702-6105, USA; 3 Austin Area Obstetrics, Gynecology and Fertility, Austin, TX 78758, USA; 4 DYSIS Medical, Edinburgh EH12 9DQ, UK; 5 Department of Obstetrics/Gynecology, University of Central Florida, Orlando, FL 32827, USA

**Keywords:** biopsy, cervical cancer, cervix uteri, cervical intraepithelial neoplasia, colposcopy, dynamic spectral imaging

## Abstract

The present study compared two methods for the detection of severe cervical dysplasia in women with atypical squamous cells of underdetermined significance (ASC-US) cytology; digital colposcopy with adjunctive dynamic spectral imaging (DSI) and conventional colposcopy. IMPROVE-COLPO was a two-arm cross-sectional study of US community-based colposcopy. The active (prospective) arm of this study recruited patients examined by digital colposcopy and adjunctive DSI. Preceding consecutive patients that had been examined with conventional methods were used as historical controls in the retrospective arm of the study after being matched in number to those in the prospective arm by a colposcopist. In the present study, the primary measure was the number of women detected with cervical intraepithelial neoplasia (CIN) grade 3 or worse (CIN3+) following punch biopsy. The study included 1,353 retrospective and 1,226 prospective patients eligible for this analysis who were examined by 146 colposcopists in 42 community-based clinics. The patient baseline characteristics were comparable between the two arms. The average number of biopsies taken per patient was higher among the prospective arm patients (including standard and DSI-assisted biopsies) compared with the retrospective arm control patients (1.21 vs. 0.97 respectively). Biopsy detected 31 patients with CIN3+ [2.29%; 95% confidence interval (CI), 1.56-3.24] in the retrospective arm, and 48 patients with CIN3+ (3.92%; 95% CI, 2.90-5.16) in the prospective arm. The difference in the number of patients detected with CIN3+ in the two arms of the study was 1.62% (95% CI, 0.30-3.04; P=0.022), which corresponds to a 70.9% relative increase in the prospective compared with the retrospective arm. Biopsy appeared less efficient in detecting patients with CIN3+ in the retrospective arm compared with the prospective arm. However, there was no statistically significant difference between the retrospective arm and the prospective arm in terms of: i) Biopsies taken (over the entire population) per patient detected with CIN3+ (42.2 in the retrospective arm vs. 30.8 in the prospective arm; P=0.164) and ii) positive predictive value of using biopsies to identify patients with CIN3+ (2.83 vs. 3.92; P=0.118). Adoption of digital colposcopy with DSI increased the number of biopsies collected from ASC-US patients compared with retrospective controls of standard colposcopy and detected a significantly higher number of patients who were CIN3+. The number of additional biopsies taken in the prospective arm compared with the retrospective arm was too small to explain the increased detection of patients with CIN3+ observed in the prospective arm, suggesting that biopsies in the prospective arm were better at identifying CIN3+.

## Introduction

The increase in cervical cancer screening intervals in the US (1) triggered concerns about increasing the risk for cancer (2) and the implementation of guidance across the US (3). Adoption of guidelines in community-based clinics is lagging and is inefficient (4). Colposcopy with biopsy is a critical link between screening and diagnosing/treating cancerous or pre-cancerous lesions; however its sensitivity, accuracy and reproducibility are limited (5–8), and the lack of standardization of colposcopic practice in the US is a recognized contributor to this (9). The American Society for Colposcopy and Cervical Pathology (ASCCP) recently published optional standards to improve the performance of colposcopy (10). Colposcopic biopsy practice varies widely, ranging from identifying and sampling the single most suspicious site, to 4-quadrant biopsies including multiple non-targeted/random biopsies from each patient (11). Based on the findings of the National Cancer Institute (NCI) Biopsy Study (12), the ASCCP recommends taking multiple targeted biopsies from patients (typically 2–4), except from patients with a low-grade referral and nothing observable at colposcopy (10). This strategy has not been validated in wider populations, and generalizing a recommendation based on academic research data to patients with a potentially low risk of cervical disease may have a negative impact on patients, such as increasing pain, discharge or bleeding from additional biopsies, unnecessarily (13). Furthermore, even with multiple biopsies, a large number of precancers are un/under-detected, indicating that punch biopsy placement is inaccurate (14). Since studies are often performed by well-trained and experienced colposcopists and follow specific protocols, in US community-based clinics, where colposcopists may be more inexperienced and don't adhere to guidelines fully (15), the adoption of multiple biopsy protocols (10) could introduce a big negative impact on patients. Screening and management recommendations are generally not fully adopted in community-based practice (15,16) and the proposed measures (10) do not address the fundamentally subjective nature of colposcopy. Subsequently, standardization of the imaging process during the colposcopic procedure, and determination of how observations are assessed and interpreted could be helpful.

Adjunctive Dynamic Spectral Imaging (DSI) mapping is a standardized way to quantify cervical acetowhitening for colposcopic assessment and biopsy selection, which increased the sensitivity of high-grade lesion detection in previous studies (17–20). Following the aforementioned studies assessing this technology, IMPROVE-COLPO was the first study to evaluate colposcopy with DSI on a US population (21,22). Furthermore, in contrast to the aforementioned previous studies, the IMPROVE-COLPO study was conducted in community-based clinics, and included a control group to allow assessment of this technology in routine practice and its comparison with the prior standard-of-care for the first time. Previous published analyses of the IMPROVE-COLPO study reported that detection of high-grade (grade ≥2) cervical intraepithelial neoplasia (CIN2+) increased with the use of DSI (21,22). In particular, for patients with an indication of low grade abnormality in screening, it was reported that a 21% increase in the number of biopsies, combined with more efficient biopsy technique, resulted in a 31 and 56% increase in the detection of women with CIN2+ and CIN3+, respectively (21).

To the best of our knowledge, the present analysis was the first to present results collected from the recruitment of 7,555 patients in total. The present study evaluated the impact of digital colposcopy combined with DSI on a well-defined sub-population, specifically the cohort of women recruited in IMPROVE-COLPO with cytology of atypical squamous cells of undetermined significance (ASC-US). The present study may be the largest conducted on this specific cohort, with this prevalent but equivocal cytologic indication representing ~1/3 of women having colposcopy (16) and being considered as relatively low-risk for high-grade disease (1,23). The present study may therefore prove valuable in improving the understanding of colposcopic practice and outcomes, as the perceived low-risk for the aforementioned patient cohort may be affecting how colposcopy is being practiced, such as how many patients are biopsied or how many biopsies are being taken, and eventually impairing the capacity of colposcopy to efficiently detect disease in this substantial patient cohort. In order to ensure a focus on clinically important disease (i.e., disease with a considerable potential to progress to cancer), and since CIN2 histology results can be ambiguous (24), all primary analyses in the present study have been performed for histological outcomes of CIN3+.

## Materials and methods

### 

#### Patient recruitment

IMPROVE-COLPO (NCT 02185599) was an observational, two-arm, cross-sectional study that recruited patients who had been referred for colposcopy across multiple US community-based clinics (21,22). The IMPROVE-COLPO study was approved by a central Institutional Review Board (IRB), E&I Review Services (Independence, USA), the University of Toledo Biomedical IRB (Toledo, USA) and the Advocate Health Care IRB (Downers Grove, USA) and was conducted according to the International Conference on Harmonization Guideline for Good Clinical Practice.

The DSI method, which is integrated as an adjunct into a commercially available digital colposcope (DYSIS; DYSIS Medical Ltd.), standardizes colposcopic imaging, quantifies and maps the acetowhitening to introduce objectivity, and supports assessments and biopsy decisions (17,18). US facilities that adopted the DSI technology participated in the present study and recruited consecutive patients undergoing colposcopy with the DSI digital colposcope for inclusion in the prospective arm. Control cases were collected by chart review from consecutive historical examinations (retrospective arm) performed at each facility by the same colposcopists as in the prospective arm, in the period directly preceding the study device installation at each facility, but with standard colposcopes and methods to capture how colposcopy is performed in a real-world setting and to characterize the standard-of-care performance. To reduce bias from variabilities among colposcopists, the number of patients recruited per colposcopist was equal in the two arms. For practical reasons, the requirement to exactly match the patient numbers per colposcopist did not include the referral reason of the patients, and therefore, the number of patients in each referral sub-category did not exactly match.

The colposcopists that participated in the present study were those performing colposcopy routinely in each facility, and comprised gynecologic oncologists, obstetrician-gynecologists, nurse practitioners and physician assistants. Colposcopists were all adequately trained for the use of the digital colposcope and interpretation of the DSI map prior to recruiting prospective patients. The inclusion criteria for patients across both arms included age ≥21 years, an abnormal screening result based on guidelines (1,25) and their ability to provide informed consent. The present study specifically focused on the sub-group of women recruited in the IMPROVE-COLPO study with an ASC-US cytologic result. To be eligible, ASC-US had to either be combined with positivity to human papillomavirus (HPV+) or be persistent at repeated screenings (23). The exclusion criteria for both arms were as follows: Lack of specified indication; a single ASC-US result without HPV positivity; pregnancy; human immunodeficiency virus infection and acquired immune deficiency syndrome positivity; previous hysterectomy; and patients receiving or having received radiation treatment or chemotherapy for cervical neoplasia or other concurrent cancer. There were 1,353 and 1,226 women included in the retrospective and prospective arms, respectively. Patients from the prospective arm signed informed consent prior to the study, whereas consent was waived by the aforementioned IRBs for patients from the retrospective arm.

#### Prospective arm (DSI)

**Figure 1.**
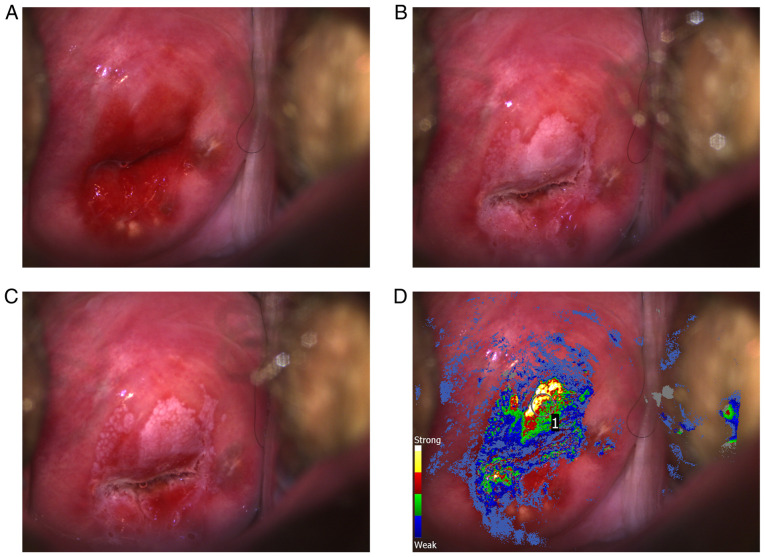
Colposcopic images from a 31-year-old non-Hispanic white/Caucasian patient after a screening result of atypical squamous cells of undetermined significance and human papillomavirus infection. (A) Image of the cervix prior to application of acetic acid (5%). (B) Image of the cervix 30 sec after acetic acid application. (C) Image of the cervix 2 min after acetic acid application. (D) Image of the cervix with the associated DSI map overlay. Extensive acetowhite lesions can be seen in (B and C), with acetowhitening of varying intensity and persistence, as well as mosaicism. Prior to observing the DSI map (D), colposcopic impression was low-grade changes and a single biopsy was selected at 12 o'clock (gray circle with number ‘1’). However, that area, as well as another area at 7 o'clock, were highlighted in the DSI map and suggested high-grade acetowhitening changes (red-yellow-white). Despite guidance for multiple biopsies in such cases, no additional biopsies were taken from this patient. The result from the biopsy was CIN3+ and the patient had subsequent loop electrosurgical excision procedure which was also CIN3. DSI, dynamic spectral imaging; CIN, cervical intraepithelial neoplasia.

The DSI digital colposcope was used for all examinations in patients in the prospective arm. This is a high-resolution digital colposcope offering DSI mapping (17), a method that is based on the analysis of a baseline image and a series of consecutive images captured for 2–3 min following acetic acid application. Acetowhite changes are quantified and highlighted for assessment and directed biopsy with a color scale (Fig. 1). Previous studies revealed that the upper part of the scale (red-yellow-white) is associated with high-grade histological findings (17,18), and may therefore be used for biopsy selection. Since the DSI map is an aid, the colposcopists could only see it after completing a thorough visual standard assessment of acetowhitening and morphology, and after documenting their biopsy selections based on visualization (standard biopsies). For patients where the colposcopist did not select any areas to biopsy based on visualization (i.e., before seeing the DSI map), this was explicitly documented. Colposcopists could use the DSI map for selecting additional biopsies at different locations, but standard biopsies should not be disregarded based on the DSI map result.

Colposcopists were responsible for all clinical decisions, ensuring that the study would capture the pragmatic, rather than an investigational, use of DSI. Decisions regarding which women required a biopsy, and the number and location of the biopsies, were not protocol-dictated. For each patient, the DSI map was interpreted and followed or ignored for biopsy selection at the colposcopists' discretion.

#### Retrospective arm (standard colposcopy control)

In order to collect a matched control group (retrospective arm of the study), appointment and billing information was used to identify consecutive patients for each colposcopist. Identified patients were subsequently selected according to study inclusion/exclusions. Data were extracted from the patient charts recorded at each clinic.

#### Data collection

Prospective arm recruitment occurred between September 2014 and October 2017. Patients in the retrospective arm (control examinations) were between November 2004 and October 2017, with >96% of them between January 2012 and October 2017 and 80% between January 2014 and October 2017. The data collected comprised basic demographics information, numbers of biopsies taken, indication whether endocervical sampling (ECS) or treatment was performed, and histopathology results. Biopsy results are presented at the single biopsy level, with a few exceptions when multiple biopsy samples had been processed and reported together, in which case they were excluded from any biopsy-level analysis. Biopsies were labeled as ‘standard’, ‘random’ or ‘DYSIS’ (for prospective patients). Histopathology readings, which are the gold standard for analyses, were performed by the laboratories used by the participating facilities, following routine practice.

Although the primary end-point of the IMPROVE-COLPO study was the detection of women with CIN2+, the current study presents an analysis primarily for detection of women with CIN3+, which is a more reliable surrogate for cervical cancer (26) compared with CIN2, which is a more ambiguous histological finding (24) and often regresses (27). In order to better understand the impact of digital colposcopy with DSI, the number of biopsied women and the number of biopsies were also analyzed and compared. Performance was compared across the two arms and, in secondary analyses, within the prospective arm (to evaluate the contribution of standard-directed vs. DSI-assisted biopsies).

#### Data analysis

Given the observational design of the IMPROVE-COLPO study and the non-investigational colposcopy (in both arms), where no biopsies or excisional treatments (loop electrosurgical excision procedure or conization) outside routine care were requested, the sensitivity of colposcopy and colposcopic biopsy to detect patients with high-grade cervical disease could not be measured because the number of subjects with undetected disease was unknown. For analyses, the ‘disease detection rate’ was used, which was defined as the number of patients with CIN3+ divided by the total number of patients. Rates were first calculated for all the detection methods combined (biopsy/ECS/treatment) as an overview. Subsequently, the detection rates were calculated, analyzed and compared specifically for colposcopic (punch) biopsies. In the retrospective arm, the detection results were also calculated for different time periods. As an indirect measure of specificity of colposcopic biopsy, the proportion of women that underwent biopsy without their biopsies revealing CIN3+ was determined. As indicative measures of biopsy efficiency in each arm, the total number of biopsies taken (over the entire population in each arm) per each patient detected with CIN3+ was calculated, and the positive predictive value (PPV) of biopsy was determined, which corresponds to the proportion of biopsies that identified CIN3+.

#### Statistical analysis

All statistical analyses were pre-planned using two-sided 5% Type I error. A two-sided Fisher exact test with a two-sided confidence interval (CI) for the difference was used to compare detection rates. A two-sided Kruskal-Wallis test was used to compare the distribution of the number of biopsies and the number of positive biopsies. An exact binomial test was used to evaluate the incremental gain in the detection rate attributed to DSI within the prospective arm.

## Results

### 

#### Patient recruitment

**Figure 2.**
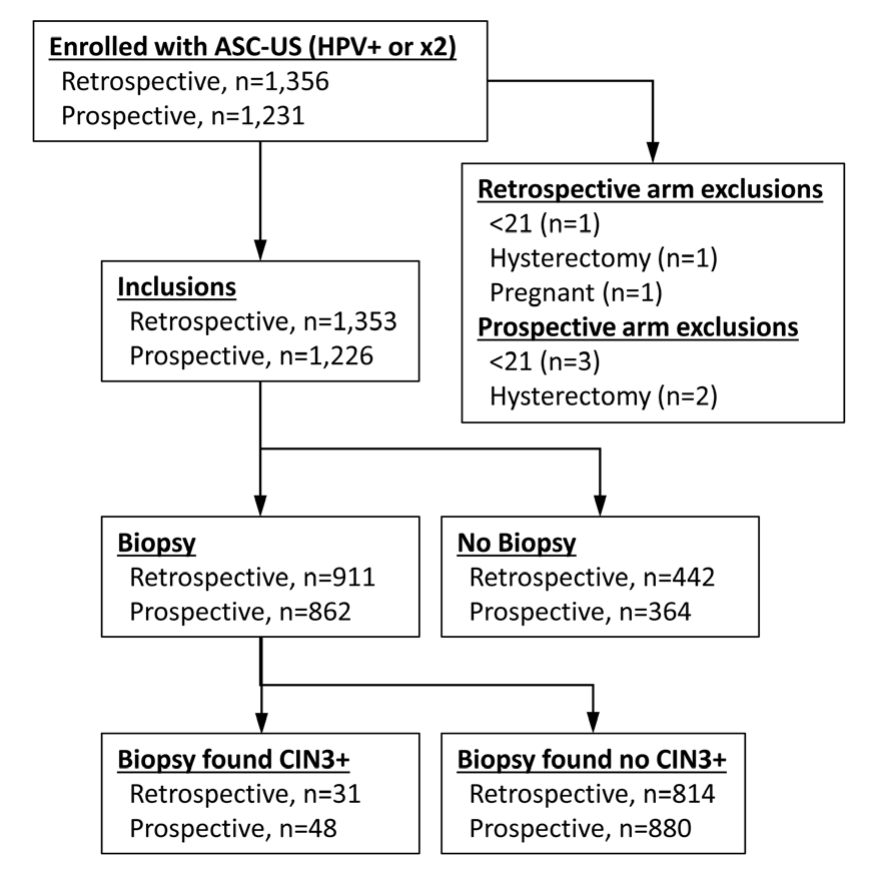
Chart describing the patient flow for the retrospective and prospective arms of the present study. The chart presents the inclusions and exclusions numbers, the number of patients that underwent biopsy and the number of patients that were diagnosed with CIN3+ following biopsy. CIN, cervical intraepithelial neoplasia.

IMPROVE-COLPO recruited a total of 7,555 women across its two arms. Among these, 2,587 (34.24%) patients had an ASC-US result and were considered for the present study. These patients were examined by 146 colposcopists in 42 clinics. Data were collected separately for each site and colposcopist, so there was no overlap of recruitment between the two arms at each clinic. No study/study-device related adverse events were reported. Eight patients were excluded for the following reasons: Age <21 years (n=4); history of hysterectomy (n=3); and pregnancy (n=1). A total of 1,353 and 1,226 women were included in the retrospective and prospective arms, respectively (Fig. 2). The majority of these patients (~93% in each arm) had an ASC-US/HPV+ referral rather than persistent ASC-US. These two sub-groups were therefore analyzed together. Patient baseline characteristics are presented in Table I. The median age of patients was 34 years in both arms. There was no significant difference for each characteristic between the two groups, apart from Hispanic ethnicity (P=0.037; two-sided Fisher's exact test).

The clinical characteristics of patients and the distribution of patients according to their detected disease status are presented in Tables SI and SII. The average and median patient ages in the two arms were comparable for patients in all histological diagnosis groups (Tables SI and SII). The percentages of patients that were biopsied (67.3% in the retrospective and 70.3% in the prospective arm; Tables SI and SII, respectively) and of patients that underwent ECS (74% in the retrospective and 73.2% in the prospective arm; Tables SI and SII, respectively) were not significantly different (P=0.106 and P=0.687, respectively; two-sided Fisher exact test). More women in the prospective arm compared with women in the retrospective arm were treated by excision (11.4 vs. 9.3%, respectively; Tables SII and SI, respectively), although this difference was not significant (P=0.080; two-sided Fisher exact test). In both arms, the number of biopsies taken per patient increased with worsening histology result (Tables SI and SII), and this was consistently higher among prospective patients compared with retrospective patients for all histological grades. Notably, most often there was no record of a colposcopic impression in the retrospective patient charts (available for 533/1,353 cases; Table SI). Conversely, the DSI software specifically required that a colposcopic impression should be recorded for each examination, so these data were available for most patients (1,164/1,226; Table SII).

In total, 126 patients with CIN3+ were detected: 56 patients in the retrospective arm (4.14%) and 70 patients in the prospective arm (5.71%) (Tables SI and SII). These patients included 4 (retrospective) and 7 (prospective) cases of adenocarcinoma *in situ* and 1 case of microinvasive squamous carcinoma (prospective arm).

Punch biopsy detected 31 women with CIN3+ (55.36% of all women detected with CIN3+) in the retrospective arm vs. 48 women with CIN3+ (68.57% of all women detected with CIN3+) in the prospective arm. ECS was performed on >73% of the patients in both arms (Tables SI and SII) and detected an additional 9 (16.07%) patients with CIN3+ in the retrospective vs. 7 (10%) in the prospective arm. A further 16 (28.57%) patients in the retrospective arm vs. 15 (21.43%) in the prospective arm were detected with CIN3+ in excisions (Tables SI and SII) that were performed on women that had first been detected with <CIN3 on biopsy/ECS.

#### Disease detection using biopsy in the two arms

In the retrospective arm, 911/1,353 women (67.3%) underwent biopsy vs. 862/1,226 women (70.3%) in the prospective arm (P=0.1062; two-sided Fisher exact test; Tables SI and SII). The total number of biopsies taken was 1,308 in the retrospective arm vs. 1,478 in the prospective arm (average per patient of 0.97 and 1.21, respectively; P<0.001; two-sided Fisher exact test) (Tables SI and SII). The number of biopsies marked as ‘random’ was small (7 in the retrospective arm and 30 in the prospective arm, Tables SI and SII).

The average number of biopsies specifically among the sub-group of patients that underwent biopsy was 1.44 (retrospective) vs. 1.71 (prospective) (Tables SI and SII). This 0.27 difference (P<0.001; two-sided Fisher exact test) is a relative increase of 18.8% (data not shown). Hence, ~1 additional biopsy per every 5 biopsied women was performed in the prospective arm.

Colposcopic biopsy detected 31 women with CIN3+ in the retrospective arm vs. 48 in the prospective arm. The detection rates were 2.29% (95% CI, 1.56-3.24) vs. 3.92% (95% CI, 2.90-5.16) respectively (Table II). The 1.62% difference (95% CI, 0.30-3.04; P=0.022; two-sided Fisher exact test) demonstrated that detection was 70.9% higher in the prospective arm (a 1.71 ratio; 95% CI, 1.1-2.66).

The difference in the detection of patients with CIN3+ in the two arms remained significant (P=0.027; two-sided Fisher exact test), also when the additional patients with CIN3+ detected by excision (12 in each arm) after biopsy/ies of CIN2 (and ECS of <CIN2) were taken into account. The detection of patients with CIN3+ was consistently higher in the prospective arm for different age sub-groups (Table II): 3.87% for women 21–24 years old (n=165), 1.20% for women 25–29 years old (n=299) and 1.41% for women >29 years old (n=889). Random biopsy detected one patient with CIN3+ (in the prospective arm).

To indirectly assess the specificity of biopsy, the number of women without CIN3+, specifically among biopsied patients, was analyzed. Of the 911 biopsied women in the retrospective arm, 880 (96.6%) had no CIN3+ biopsy vs. 814 of the 862 biopsied women (94.42%) in the prospective arm, a 2.17% difference (P=0.029; two-sided Fisher exact test; data not shown).

Detection in the retrospective arm was analyzed for the different time periods (data not shown), although patient numbers for the earlier years was too small for a definitive conclusion. For the 50 women (3.7% of the total) examined between 2004 and 2011, before ASCCP screening guidelines (1) were published and when conventional cytology may have been the primary approach (compared with liquid-based cytology), detection rate was 2% (95% CI, 0.05-10.65%). Between 2012 and 2017 (1,303 women, 96.3% of the total), the detection rate was 2.30% (95% CI, 1.56-3.27%). Between 2014 and 2017 (1,078 women, 79.7% of the total), detection rate was 2.41% (95% CI, 1.58-3.51%). These findings were consistent with the overall result of 2.29%.

In a secondary analysis, CIN2+ detection was investigated, and results confirmed a similar trend as with CIN3+, as there were 91 women in the retrospective and 116 in the prospective arm (including 1 detected by random biopsy), which correspond to detection rates of 6.73% (95% CI, 5.45-8.19%) and 9.46% (95% CI, 7.88-11.24%) respectively, a 2.74% difference (95% CI, 0.55-4.93%; P=0.011; two-sided Fisher exact test) and a 40.7% increase in the prospective arm compared with the retrospective arm (data not shown).

#### Incremental disease detection with DSI

Among the 1,226 patients from the prospective arm, the incremental detection of patients with CIN3+ diagnosed by additional DSI-assisted biopsies selected at different locations on the cervix after visual assessment biopsies was analyzed (data not shown). Overall, 659 (53.8%) patients had standard biopsies, and 190 (15.5%) of these patients also had DSI-assisted biopsies. In addition, 196 patients (16%) underwent DSI-assisted biopsies without a standard biopsy, whereas an additional 7 patients (0.6%) underwent random biopsy only and 364 patients (29.7%) had no biopsy taken (data not shown).

Altogether, there were 966 standard biopsies and 482 DSI-assisted biopsies (Table SII). Standard visual assessment biopsy in the prospective arm (a biopsy selected prior to seeing the DSI map) detected 30 patients with CIN3+ (detection rate 2.45%; 95% CI, 1.66-3.48%), which is comparable to the 2.29% rate observed in the control group. DSI-assisted biopsies detected 17 additional patients with CIN3+, increasing the rate to 3.83% (95% CI, 2.83-5.07%), a 1.38% difference that is statistically significant (two-sided P=0.006; exact binomial test relative to a 2.45% null hypothesis) and a 56.3% relative increase in detection for the prospective arm compared with the retrospective arm (data not shown). One additional patient with CIN3+ was detected using random biopsy; however, as there were too few random biopsies, these were not considered.

The majority of the 17 additional patients with CIN3+ that were detected by DSI-assisted biopsies selected after visual assessment, would have been missed without the use of DSI (data not shown in Tables). For 11 of these patients (64.7%), before observing the DSI map, the colposcopist had confirmed that they would not perform a biopsy; 4 patients (23.5%) had standard visual biopsies that were negative or CIN1 and 2 patients (11.8%) had CIN2 in a standard visual biopsy preceding the DSI-assisted biopsy that detected CIN3+.

A similar trend was observed for the detection of women with CIN2+. Standard visual biopsy detected 75 women, which corresponds to a detection rate of 6.12% (95% CI, 4.84-7.61%). The additional DSI-assisted biopsies detected another 40 women with CIN2+, which increased the detection rate to 9.38% (95% CI, 7.81-11.15%), a 53.3% relative increase over the detection by standard visual biopsy.

#### Biopsy efficiency

In a secondary analysis, the biopsy efficiency to detect CIN3+ was investigated, and the results were compared between the two arms and within the prospective arm (data not shown).

Comparing the total number of biopsies taken in each arm to the number of patients detected with CIN3+, suggests that biopsy was less efficient in the retrospective arm, i.e., more biopsies were required to detect a patient with CIN3+ in the retrospective compared with the prospective arm (42.2 vs. 30.8, respectively); however, this difference was not statistically significant (two-sided P=0.164 for the ratio, computed by inverting the two exact binomial tests).

To compare the PPV of biopsy in the two arms, 1 patient with CIN3 from the retrospective arm was excluded as she had multiple biopsies taken, but all of them had been processed together. CIN3+ was detected in 37/1,306 biopsies in the retrospective arm vs. 58/1,478 in the prospective arm. The PPVs were 2.83% (95% CI, 2.0-3.88%) and 3.92% (95% CI, 2.99-5.04%) in the retrospective and prospective arms, respectively. The 1.09% difference was not statistically significant (95% CI, −0.27-2.46%; P=0.118; two-sided Fisher exact test).

Within the prospective arm, biopsies were considered separately as standard/visual and DSI-assisted, in order to analyze their PPV. Overall, 34/966 standard biopsies and 23/482 DSI-assisted biopsies were CIN3+, with PPVs of 3.52% (95% CI, 2.45-4.88%) and 4.77% (95% CI, 3.05-7.07%), respectively. The 1.25% difference was not statistically significant (95% CI, −0.83-3.75%; P=0.254; two-sided Fisher's exact test).

To compare standard/visual biopsy across the two arms, only the first part of prospective arm examinations (biopsy selection by standard visualization) was considered. The number of standard/visual biopsies per patient was higher in the retrospective arm compared with the prospective arm (0.97 vs. 0.79; P<0.001; two-sided Kruskal-Wallis test); however, the PPV for detecting CIN3+ was comparable to prospective arm biopsies (2.83% vs. 3.52%; P=0.394; two-sided Fisher's exact test), resulting in the comparable detection rates for CIN3+ (2.29% vs. 2.45%; P=0.797; two sided Fisher's exact test).

## Discussion

ASC-US is an equivocal cytologic state with low-risk for CIN3+ (6), unless it is combined with HPV positivity or persistence. In this case, the risk matches the risk of low-grade squamous intraepithelial lesion (LSIL), leading to a referral for colposcopy (23). The present analysis of patients with ASC-US demonstrated that the use of the study's digital colposcope and the adjunctive DSI aid, resulted in a CIN3+ detection rate significantly higher compared with retrospective controls, which was achieved with only 1 more biopsy per 5 patients and without increasing the number of women undergoing biopsy. The increased number of biopsies in the prospective arm appears insufficient to explain the increased detection of patients with CIN3+, as the 18.8% increase in the number of biopsies increased the detection by as much as 70.9%.

Biopsies selected based on standard visualization (DSI-assisted biopsies not considered for the prospective arm) had comparable detection rates (2.29 vs. 2.45%), indicating that visual/standard colposcopy may be equivalent in the two arms and that the overall increase in the prospective arm may have been primarily due to the use of adjunctive DSI for biopsy selection. The secondary analyses performed on the biopsy-level did not find significantly different results, which may be due to insufficient numbers, and should therefore be interpreted with caution. However, the results from these secondary analyses suggested that prospective arm biopsies were more efficient than retrospective control arm biopsies. Proportionally more biopsies detected CIN3+ and it took fewer biopsies to identify a patient with CIN3+ in the prospective arm. Furthermore, the DSI-assisted biopsies specifically, exhibited the highest PPV, despite the disadvantage of being selected after the visual assessment, which should have identified the majority of obvious lesions, had been completed.

A previous analysis from the IMPROVE-COLPO study analyzed results from a less specific cohort than in the present study and used the global threshold for high-grade disease (CIN2) (21), which however, is less reproducible than CIN3 and often regresses (27). This previous study analyzed together all low-grade referrals, i.e., women with LSIL, ASC-US and HPV+. It had included about half of the women that are also included in the present analysis (1,461/2,579) and had reported that detection of women with high-grade disease (CIN2+) was 31% higher in the prospective arm compared with the controls. The association between detection of CIN2+ and numbers of biopsies taken was modelled, suggesting that biopsy efficiency was higher in the prospective arm (21), which is consistent with the observations from the present study. Another previous study that analyzed mixed referrals (low-grade and high-grade) and in particular the effect of DSI-assisted biopsies, had obtained similar conclusions (22).

Comparing populations and findings in the present study of colposcopy in community-based clinics with those of academic institution-based trials (6,12) or other large cohorts (28), highlights some differences. In the HPV triage arm of the ASC-US LSIL triage study (ALTS), 8.7% of patients with ASC-US had CIN3+ (6), and in the NCI biopsy study, ~7% of ASC-US patients had CIN3+ (12). These numbers are higher compared with the overall incidences reported in the present study (4.14 and 5.71% in the retrospective and prospective arm, respectively). The Kaiser Permanente Northern California (KPNC) reported a 5-year cumulative incidence for CIN3+ among patients with ASC-US/HPV+ of 4.95% (28), which is closer to the 4.14 and 5.71% detected in the present study in a single colposcopy visit. These disparities may be explained by differences in population characteristics. For example, in the NCI study, the median patient age was 26 years (12), whereas it was 34 years in the present study. In addition, the distribution of referral grades in these cohorts varied considerably. The ASC-US cases represented 23.8% in the NCI biopsy study, 20.9% in KPNC and 34.2% in the present study. Although this is difficult to interpret, it may be hypothesized that the considerably higher proportion of ASC-US referrals among patients having colposcopy in community-based clinics in the present study, combined with the higher age and a mostly privately insured population, may suggest that this is a lower-risk population compared with what is typically seen in academic centers. The present study suggests that the incorporation of the DSI map to identify and quantify acetowhitening is an aid that increases disease detection, supporting adherence to ASCCP guidance by ensuring that acetowhitening is not underestimated and that biopsy placement is improved.

The present study had some limitations. Firstly, without additional biopsies, excisional treatments or longitudinal follow-up, cases of missed disease cannot be confirmed, precluding the calculation of true sensitivity. However, the relative increase of detection rate in the prospective arm compared with that of the retrospective arm corresponds to an equal increase in sensitivity. Without additional detailed analyses of the histological findings that were out of scope (e.g., adjudication and lesion size comparison in treatment specimen), it is hard to confirm the clinical significance of the present results. However, since the primary analysis in the present study was for CIN3+ and the difference in detection was significant, the results from this study may reflect true impact on patient outcomes. Secondly, a direct comparison between the use of DSI for additional biopsies and the collection of additional ‘standard’ biopsies per patient (10,12) was not addressed in the present observational setting. Thirdly, the lack of strict guidance by the study protocol on how to use the DSI map for biopsy could introduce uncertainty about the generalization of the results from the present study; however, this may have a minimal impact due to the large sample size and the large number of participating colposcopists.

One strength of the present study is that ‘real-world’ data, i.e., data representing routine practice, were collected both in the control arm and in the active arm with pragmatic use of DSI, from consecutive patients and with minimal exclusions. In US community-based colposcopy clinics, in contrast to recommendations (10,12), biopsy is performed conservatively (16) as illustrated by the <1 biopsy being taken per patient with ASC-US in the present study, which may be leading to disease being missed.

In conclusion, the results from the present study suggest that digital colposcopy combined with DSI mapping may increase the detection of CIN3+ lesions among women with ASC-US. The results from the present study also suggest that, in order to obtain a detection rate for standard visual colposcopy comparable to that achieved with the use of DSI mapping, colposcopists would likely have to increase the number of biopsies taken per patient by more than the 18.8% observed in the present study. These findings may allow the implementation of changes in clinical practice, and therefore reduce the negative impact on patients and the cost of medical care.

## Supplementary Material

Supporting Data

## Figures and Tables

**Table I.** Baseline characteristics of the study population.

Characteristic	Retrospective control arm	Prospective arm
Patients, n	1,353	1,226
Age, years		
Median	34.0	34.0
Average	36.5	36.7
Pre-menopausal^a^, n (%)	1,167 (86.3)	1,074 (87.6)
Post-menopausal, n (%)	185 (13.7)	185 (12.4)
Insurance status^a^, n (%)		
Private	1,212 (89.6)	1,090 (88.9)
Medicare	26 (1.9)	24 (2.0)
Medicaid/Other	95 (7.0)	83 (6.8)
Uninsured	18 (1.3)	25 (2.0)
Ethnicity^a^, n (%)		
Caucasian	822 (60.8)	737 (60.1)
African-American	311 (23.0)	269 (21.9)
Asian	33 (2.4)	31 (2.5)
Hispanic	141 (10.4)	161 (13.1)
Other	46 (3.4)	28 (2.3)
Referral pathway^a^, n (%)		
ASC-US/HPV+	1,266 (93.6)	1,137 (92.7)
Persistent ASC-US	87 (6.4)	89 (7.3)

a Cases with missing data not included. ASC-US, atypical squamous cells of undermined significance; HPV, human papillomavirus.

**Table II.** Detection rates of patients with CIN3+ identified by directed biopsy in the two study arms overall and by age group. Within the prospective arm, data are also shown separately for standard and incremental DSI-assisted biopsies.

	Retrospective control			Prospective arm					
					
Age, years	Standard Biopsy		Total	Standard biopsy		DSI-assisted biopsy	Difference^a^, %	P-value	Relative gain, %
Total, n		1,353			1,226				
Detection rate, %	2.29		3.92^b^	2.45		1.39	1.62	0.022	70.9
21-24									
Patients, n		165			118				
Detection rate, %	1.21		5.08	3.39		1.69	3.87	0.071	319.5
25-29									
Patients, n		299			261				
Detection rate, %	3.01		4.21	3.07		1.15	1.20	0.498	40.0
>29									
Patients, n		889			847				
Detection rate, %	2.25		3.66^b^	2.13		1.42	1.41	0.089	62.7

a Differences are between detection rates in the retrospective control arm and the total of the prospective arm with P-values calculated using a two-sided Fisher's exact test

b Total includes a 36-year-old CIN3+ patient identified by Random biopsy in the prospective arm. CIN, cervical intraepithelial neoplasia; DSI, Dynamic Spectral Imaging.

## Data Availability

All data generated or analyzed during this study are included in this published article.

## Abbreviations

ASCCP: American Society for Colposcopy and Cervical Pathology
NCI: National Cancer Institute
DSI: dynamic spectral imaging
CIN: cervical intraepithelial neoplasia
ASC-US: atypical squamous cells of underdetermined significance
IRB: Institutional Review Board
HPV: human papillomavirus
ECS: endocervical sampling
PPV: positive predictive value
CI: confidence interval
LSIL: low-grade squamous intraepithelial lesion
ALTS: ASC-US LSIL triage study
KPNC: Kaiser Permanente Northern California
