# Supplementary material for: Rate of detecting CIN3+ among patients with ASC-US using digital colposcopy and dynamic spectral imaging
Source: Oncol Lett. 2020 Jul 16;20(4):17. doi: 10.3892/ol.2020.11878 (PMC7406885; doi:10.3892/ol.2020.11878)
Supplement: Supporting Data [file Supplementary_Data.pdf]

Table SI. Clinical characteristics of the patients in the retrospective control arm.

		Histological diagnosis[a]						
Characteristic	Total, n	<CIN1	CIN1	CIN2	CIN3	CGIN2-3	Invasive	CIN3+
Patients, n (%)	1,353 (100.0)	854 (63.1)	381 (28.2)	62 (4.6)	52 (3.8)	4 (0.3)	0	56 (4.1)
Median age, years	34.0	34.0	34.0	30.0	32.5	35.0	NA	32.5
Average age, years	36.5	37.2	35.6	33.8	35.3	44.5	NA	35.9
Colposcopic impression[b], n (%)								
Normal	292 (54.8)	237 (70.7)	41 (27.5)	5 (22.7)	9 (36.0)	0	0	9 (33.3)
Low-grade lesion	216 (40.5)	93 (27.8)	100 (67.1)	9 (40.9)	12 (48.0)	2 (9.1)	0	14 (51.9)
High-grade lesion	25 (4.7)	5 (1.5)	8 (5.4)	8 (36.4)	4 (16.0)	0	0	4 (14.8)
Biopsy								
Biopsies taken	1,308	627	517	89	72	3	0	75
Biopsied patients, n (%)	911 (67.3)	463 (54.2)	341 (89.5)	57 (91.9)	48 (92.3)	2 (50.0)	0	50 (89.3)
Average biopsies	1.44	1.35	1.52	1.56	1.50	1.50	NA	1.50
Standard biopsies	1,301	623	516	89	72	1	0	73
Random biopsies	7	4	1	0	0	2	0	2
ECS								
Patients with ECS, n (%)	1,001 (74.0)	604 (70.7)	302 (79.3)	51 (82.3)	40 (76.9)	4 (100.0)	0	44
Worst result by ECS, n	276	224	37	7	5	3	0	8
Treatment[b], n (%)								
Patients with LEEP/cone	126 (9.3)	6 (0.7)	31 (8.1)	41 (66.1)	47 (90.4)	1 (25.0)	0	48
Worst result by LEEP/cone	41	3	17	5	15	1	0	16
Cryotherapy	36 (2.7)	1 (0.1)	26 (6.8)	7 (11.3)	2 (3.8)	0	0	2 (3.6)
Hysterectomy	9 (0.7)	4 (0.5)	2 (5.9)	1 (3.3)	2 (6.2)	0	0	2 (6.2)

[a]Worst diagnosis overall (including patients detected by biopsy, endocervical sampling or excisional treatment). [b]Cases with missing data not included. CIN, cervical intraepithelial neoplasia; CGIN, cervical glandular intraepithelial neoplasia; ECS, endocervical sampling; LEEP, loop electrosurgical excision procedure; NA, not applicable.

Table SII. Clinical characteristics of the patients in the prospective arm.

		Histological diagnosis[a]						
Characteristic	Overall	<CIN1	CIN1	CIN2	CIN3	CGIN2-3	Invasive	CIN3+
Patients, n (%)	1,226 (100.0)	723 (59.0)	367 (29.9)	66 (5.4)	62 (5.1)	7 (0.6)	1 (0.1)	70 (5.7)
Median age, years	34.0	35.0	34.0	34.0	31.0	29.0	45.0	31.0
Average age, years	36.7	37.1	36.7	35.0	34.5	29.7	45.0	34.1
Colposcopic impressionb, n (%)								
Normal	527 (45.3)	381 (55.5)	110 (31.4)	17 (29.3)	17 (29.3)	1 (14.3)	1 (100)	19 (28.8)
Low-grade lesion	619 (53.2)	296 (43.1)	236 (67.4)	43 (65.5)	38 (65.5)	6 (85.7)	0	44 (66.7)
High-grade lesion	18 (1.5)	9 (1.3)	4 (1.1)	2 (3.2)	3 (5.2)	0	0	3 (4.5)
Colposcopic impression with DSI, n (%)								
Normal	239 (20.0)	198 (28.1)	33 (9.2)	4 (6.5)	4 (6.6)	0	0	4 (5.8)
Low-grade lesion	540 (45.3)	323 (45.9)	180 (50.3)	23 (37.1)	13 (21.3)	1 (14.3)	0	14 (20.3)
High-grade lesion	414 (34.7)	183 (26.0)	145 (40.5)	35 (56.5)	44 (72.1)	6 (85.7)	1 (100)	51 (73.9)
Biopsy								
Biopsies taken, n	1,478	633	602	122	107	13	1	121
Biopsied patients, n (%)	862 (70.3)	396 (54.8)	337 (91.8)	63 (95.5)	58 (93.5)	7 (100)	1 (100)	66 (94.3)
Average biopsies	1.71	1.60	1.79	1.94	1.84	1.86	1.00	1.83
Standard biopsies, n	966	410	409	73	64	10	0	74
DSI-assisted biopsies, n	482	205	184	47	42	3	1	46
Random biopsies, n	30	18	9	2	1	0	0	1
ECS								
Patients with ECS, n (%)	898 (73.2)	511 (70.7)	291 (79.3)	52 (78.8)	37 (59.7)	6 (85.7)	1 (100)	44 (62.9)
Worst result by ECS, n	238	200	30	4	4	0	0	4
Treatment[b]								
Patients with LEEP/cone, n (%)	140 (11.4)	7 (1.0)	19 (5.2)	57 (86.4)	51 (82.3)	6 (85.7)	0	57 (81.4)
Worst result by LEEP/cone, n	32	5	7	4	13	3	0	16
Cryotherapy, n (%)	33 (2.7)	1 (0.1)	30 (8.2)	0	2 (3.2)	0	0	2 (2.9)
Hysterectomy, n (%)	13 (1.1)	5 (0.7)	3 (0.8)	2 (3.0)	2 (3.2)	0	1 (100)	3 (4.3)

[a]Worst diagnosis overall (including patients detected by biopsy, endocervical sampling or excisional treatment). [b]Cases with missing data not included. CIN, cervical intraepithelial neoplasia; CGIN, cervical glandular intraepithelial neoplasia; DSI, dynamic spectral imaging; ECS, endocervical sampling; LEEP, loop electrosurgical excision procedure; NA, not applicable.
